# Diagnostics and Management of Male Infertility in Primary Ciliary Dyskinesia

**DOI:** 10.3390/diagnostics11091550

**Published:** 2021-08-26

**Authors:** Channa N. Jayasena, Anu Sironen

**Affiliations:** 1 Section of Investigative Medicine, Imperial College London, London W12 0NN, UK; c.jayasena@imperial.ac.uk; 2 Natural Resources Institute Finland, Production Systems, 31600 Jokioinen, Finland

**Keywords:** PCD, sperm, male fertility, ICSI

## Abstract

Primary ciliary dyskinesia (PCD), a disease caused by the malfunction of motile cilia, manifests mainly with chronic recurrent respiratory infections. In men, PCD is also often associated with infertility due to immotile sperm. Since causative mutations for PCD were identified in over 50 genes, the role of these genes in sperm development should be investigated in order to understand the effect of PCD mutations on male fertility. Previous studies showed that different dynein arm heavy chains are present in respiratory cilia and sperm flagellum, which may partially explain the variable effects of mutations on airways and fertility. Furthermore, recent studies showed that male reproductive tract motile cilia may play an important part in sperm maturation and transport. In some PCD patients, extremely low sperm counts were reported, which may be due to motile cilia dysfunction in the reproductive tract rather than problems with sperm development. However, the exact roles of PCD genes in male fertility require additional studies, as do the treatment options. In this review, we discuss the diagnostic and treatment options for men with PCD based on the current knowledge.

## 1. Introduction

**Figure 1:**
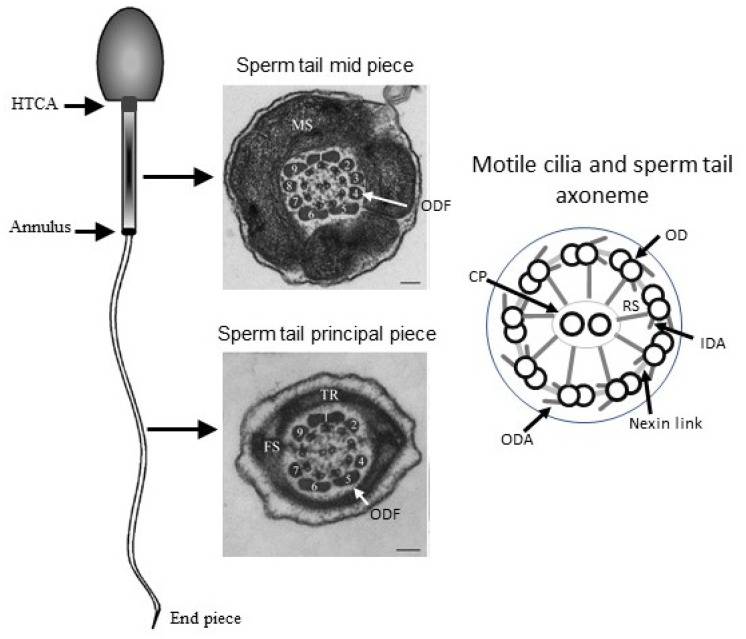
The conserved axonemal core structure is present in motile cilia and sperm flagella. The sperm tail can be divided into three parts: the midpiece, principal piece, and end piece. Outer dense fibers (ODFs) run along the mid and principal piece, and mitochondria (MS, mitochondrial sheath) surround the ODFs in the midpiece. In the principal piece, ODFs 3 and 8 are replaced by transfers ribs (TR) and form part of the fibrous sheath (FS). The annulus is a diffusion barrier between the midpiece and principal piece, and the sperm tail is connected to the head by the head-tail coupling apparatus (HTCA). The axoneme runs along the whole tail and appears ultrastructurally identical in the sperm flagellum and motile cilia, containing nine outer doublet microtubules (OD) and a central pair (CP). Radial spokes (RD) connect the ODs to the CP and nexin links connect the adjacent ODs. The head-tail coupling apparatus (HTCA) connects the sperm tail to the head and is formed by the centriole attachment to the nucleus. The annulus is a diffusion barrier between the midpiece and principal piece. The sperm illustration and TEM images are reproduced with the Creative Commons CC by license [1].

Primary ciliary dyskinesia (PCD) is caused by mutations in the genes required for motile axoneme formation and function. The axoneme is a microtubular structure consisting of nine outer doublets and two central microtubules (Figure 1).

In motile cilia, the motor force for movement is generated by outer and inner dynein arms (ODA and IDA, respectively), which are coordinated by the nexin-dynein regulatory complex (N-DRC), radial spokes (RS), and the central pair (CP) microtubules. The N-DRC complex also forms connections with other axonemal complexes within the ciliary unit and, thus, likely plays a role of a main regulatory hub [2]. These structures are also present in the sperm tail axoneme and are required for sperm motility and, therefore, for male fertility. Recent studies have identified protein components of different axonemal structures and preassembly factors [3,4,5], which can be considered as candidate genes for PCD and male infertility. However, the structure of motile cilia and sperm tails are not identical. The most obvious difference is the sperm tail length, which is 5–10 times longer than a cilium. A sperm tail contains accessory structures in addition to the core axoneme (Figure 1) and can be divided into three parts: the midpiece, principal piece, and end piece. Outer dense fibers (ODFs) run along the midpiece and principal piece, supporting the long axoneme. The principal piece contains the fibrous sheath and the midpiece contains mitochondria, which produce energy for sperm motility [6]. The sperm tail axoneme is often disintegrated when ODFs and the fibrous sheath are malformed [7,8,9]. The midpiece and principal piece are separated by a diffusion barrier called the annulus (Figure 1). The sperm tail is connected to the head through the head-tail coupling apparatus (HTCA), which is formed by the modification of the centrosomes and the formation of supporting structures to connect the tail to the implantation fossa of the nuclear membrane [10,11]. Furthermore, the axonemal protein complexes may differ in protein content between motile cilia types, which was recently demonstrated by the identification of sperm tail specific dynein heavy chains [12,13]. The different motility patterns of sperm tails and motile cilia likely originate in part from the variable composition of axonemal structures [14,15]. More research is required to establish the specific characteristics of the sperm axoneme.

## 2. Role of PCD Genes in Male Fertility

**Figure 2:**
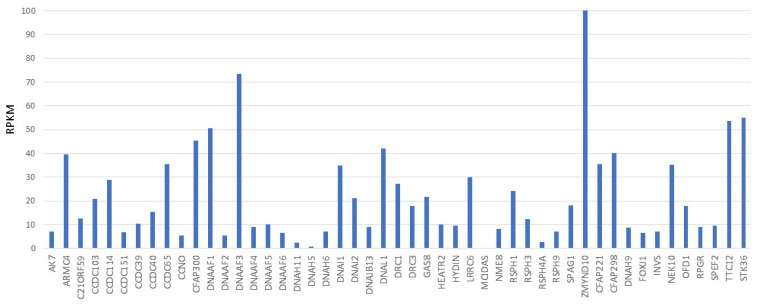
PCD gene expression in the human testis. Variable expression of known PCD genes are detected in NGS data by Fagerberg et al. [18]. RPKM reads per kilobase of transcript per million reads mapped.

Male infertility is often associated with PCD, but the pathogenic mechanisms linking PCD mutations to defective sperm function are less well understood. Thus far, mutations in approximately 50 genes have been identified as the cause of PCD, and the number of genes has rapidly increased during recent years [16,17]. Most of the PCD genes are expressed in the testis, although extremely low expression is detected for the multiciliogenesis gene *MCIDAS*, the ODA genes *DNAH5* and *DNAH11,* and the RS gene *RSPH4A* (Figure 2).

PCD genes can be divided into different categories: multiciliogenesis, dynein arm preassembly, ODA/IDA, RS/CP, and genes associated with nexin links and microtubular organization. Current knowledge on the roles of these genes in motile cilia and sperm development was recently reviewed by Shoemark and Harman [19] and Sironen et al. [20], respectively. The effects of RS/CP gene mutations on male fertility are poorly known. CP components HYDIN [1] and SPEF2 [21] cause male infertility and PCD. In addition, mutations in *SPEF2* result in a sperm-specific defect: multiple morphological abnormalities of the sperm flagella (MMAF) [22,23]. Other CP components associated with MMAF are AK7, ARMC2, and CFAP69 [24,25,26,27]. For AK7 (adenylate kinase 7), an association with PCD has also been suggested [28,29]. Male infertility was likewise reported for mutations in the RS genes *RSPH1*, *RSPH3*, and *RSPH9* [30,31,32], but, as suggested by low expression in the testis (Figure 2), RSPH4A does not seem to be required for fertile sperm production. This assumption is also supported by the fact that all reported PCD patients are fertile [31], although reported patient numbers are very low for all RS genes (number of patients 1−3). A candidate compensating gene for *RSPH4A* in the testis is *RSPH6A*, which is predominantly expressed in the testis [33]. Previous studies and expression data suggest that dynein arm preassembly genes are required for sperm motility and, therefore, mutations in these genes can be expected to cause male infertility (Figure 2, [34]), although differences in assembly mechanisms exist. A lack of dynein arm preassembly factor TTC12 results in IDA and ODA loss in sperm flagellum, but only partial loss of IDA in motile cilia [35]. This further supports the hypothesis of different dynein arm complexes in sperm and motile cilia, which has been suggested by reported variable fertility in PCD patients with mutations in genes coding for ODA [20,31,36,37].

### 2.1. Sperm Specific Dynein Heavy Chain Genes

Although the ultrastructures of motile cilia and sperm tail axoneme are similar, there are differences in the protein content of the dynein arm components. Recent studies and expression analysis (Figure 2) have shown that motile cilia dynein heavy chains *DNAH5* and *DNAH11* are not present in sperm, which suggests that mutations in these genes do not cause male infertility [12,13,20]. However, cases of infertility have been reported for patients with mutations in *DNAH5* and *DNAH11* [31,36,37]. Furthermore, mutations in *DNAH1*, *DNAH2*, *DNAH8*, and *DNAH17* have been shown to cause predominant male infertility, although mild PCD symptoms may be present [13,38,39,40,41,42,43,44,45,46,47,48,49]. Interestingly, asthenospermia without PCD symptoms was recently reported in two patients with *DNAH9* mutations [50]. *DNAH9* is a known PCD gene, which is required for distal ODA assembly in motile cilia and causes mild PCD symptoms [51,52]. The expression pattern of axonemal genes suggests cell specific differences in protein complexes, which may explain the unique motility patterns of motile cilia and sperm tails (Figure 2, [20]). However, these differences require additional research to confirm the role of DNAH genes in male fertility.

### 2.2. Male Specific Motile Cilia

**Figure 3:**
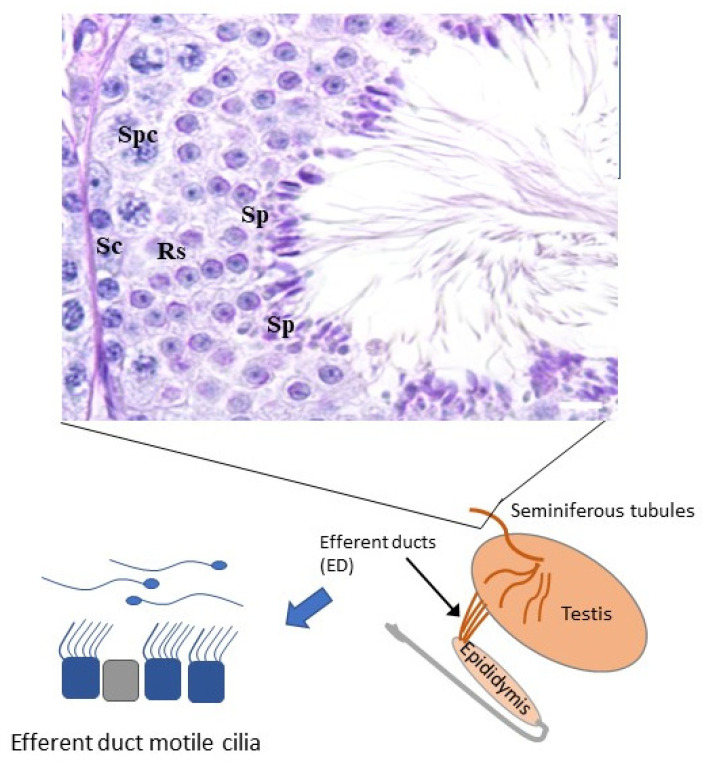
Sperm development and maturation. Male germ cells develop in seminiferous tubules of the testis. After release into the lumen of the seminiferous tubules, sperm are transported through the efferent ductules and epididymis and stored in the cauda epididymis. Efferent ductules contain motile cilia, which were shown to be crucial for male fertility in mouse models [53]. Spc = spermatocyte, Rs = round spermatid, Sc = Sertoli cell, Sp = spermatid.

Male germ cells differentiate in the seminiferous tubules of the testes and are released into the lumen of these tubules for transport to the epididymis. During this transit, sperm are concentrated and matured before being stored in the cauda epididymis for subsequent ejaculation. When sperm exit the testis, they first enter a series of thin ciliated tubules, the efferent ductules (ED, Figure 3), which connect the rete testis to the epididymis.

The ED ciliated epithelial cells are thought to create a fluid turbulence that concentrates sperm by promoting fluid absorption by neighboring non-ciliated cells [53,54]. Multiciliogenesis regulator knock-out (KO) mouse models have shown that ED cilia are required for male fertility [53] since *MCIDAS*, *CCNO* and *GEMC1* are not expressed in the testis (cells are not multiciliated, Figure 2), but are in ED. Depletion of these genes in KO mice resulted in lack of sperm in the epididymis (azoospermia) [53]. Fluid accumulation in multiciliogenesis KOs caused back pressure to the seminiferous tubules and degeneration of spermatogenesis [53]. The identified azoospermia phenotype in mice may be partially caused by defects in sperm transport and degeneration of spermatogenesis. These results suggest that genes may affect male fertility in PCD patients, although they are not required for sperm development. The roles of ED cilia in PCD patients are not known, but, in addition to sperm tail formation, it should be considered that the malfunction of ED cilia may contribute to male infertility.

## 3. Diagnosing Male Infertility in PCD Patients

### 3.1. Semen Quality Analysis

The overall prognosis of fertility in men with PCD has not been defined clearly; however, a previous study indicated that 75% of couples affected by PCD in the male partner were diagnosed as infertile [31]. Since there is a high chance of infertility in PCD patients, this information should be included in patient counselling, particularly during transition from pediatric care to adult clinics. Since there is not enough information about the roles of different PCD genes in sperm production and maturation, sperm quality should be analyzed prior to family planning. Semen analysis in fertility clinics, based on WHO guidelines (WHO, 2010), provides the initial assessment of fertility. The analysis will estimate the sperm concentration, motility, and morphology, which are used to classify the sperm phenotype (Table 1). In PCD patients, low sperm counts, decreased motility, and/or morphological abnormalities can be expected [20].

For sperm quality estimation, a sperm sample is produced after 72 h of ejaculation avoidance. At the fertility clinic, medical history and information about any factors influencing fertility will be collected. Testicular size and hormonal levels are evaluated in addition to sperm quality. Sperm quality estimates include sperm count, volume, head and tail morphology, total and progressive motility, and pH. The correct pH, which is 7.2–7.8 according to the WHO, is important for sperm motility and capacitation [55]. Decreased fertility can be expected if values are below normal (Table 2).

In the case of reduced fertility, assisted reproductive technology (ART) can be considered based on sperm quality analysis. Although semen microscopic analysis is the first stage of diagnosis, molecular testing is needed to more precisely evaluate the sperm quality, especially prior to ART.

### 3.2. Sperm Viability

Semen analysis provides a general view of the sperm quality, but in the case of immotile sperm in PCD patients, it is also important to assess the sperm viability prior to the use of ART. Fertilization rates using immotile sperm are significantly lower than with motile sperm, underlining the importance of identification of good-quality sperm [56]. Use of immotile or malformed sperm for fertilization may increase the likelihood of selecting nonviable sperm as has also been suggested by sperm viability and fertilization studies in PCD patients [57,58]. The human sperm viability can be analyzed using various methods: chemical sperm activation, light microscopy including eosin staining, hypo-osmotic swelling test (HOST), Sybr-14/propidium iodide assay, sperm tail flexibility test (STFT), and laser-assisted immotile sperm selection (LAISS). The methods assess the plasma membrane integrity, which also plays an important role in sperm capacitation, acrosome reaction, hypermotility, and sperm fusion with the oocyte.

HOST: sperm is placed in a hypo-osmotic medium, where viable sperm tails curve or swell. These spermatozoa can be selected for fertilization and washed to regain their normal shape. HOST was shown to increase fertilization rates from 30% to 44% in fresh testicular sperm and from 26% to 43% in frozen testicular sperm [59]. The method has also been successfully employed in PCD patients, with several successful pregnancies resulting [57,60,61,62]. The limitations with HOST are the high false positive rate and that it is not suitable for cryopreserved and processed ejaculated sperm.

Sperm activation: chemical sperm activation can be induced by pentoxifylline or theophylline and responsive sperm selected for use in ART. The pentoxifylline treatment was shown to be significantly more effective than HOST for identification of spermatozoa in terms of fertilization (62% vs. 41%) and clinical pregnancy rates (32% vs. 16% [63]). PCD sperm is often unresponsive to chemical activation, but pentoxifylline was successfully used to activate ejaculated spermatozoa prior to fertilization, resulting in viable pregnancy [64]. However, the use of sperm activation is limited in PCD sperm and the safety of the chemical compounds should be considered.

STFT: the sperm tail flexibility test is based on a simple principal that the viable sperm tail can be bent by a mechanical force. STFT can be very reliable and similar fertilization rates with testicular immotile or motile sperm (66% vs. 74% frozen and 73% vs. 64% fresh, respectively), and comparable pregnancy and healthy baby rates were reported [65]. STFT is safe for the developing embryo, as there are no additives, and it is simple and quick to perform, although it requires highly experienced personnel. This method has been successfully used in PCD patients [66].

LAISS: in laser-assisted immotile sperm selection, a single laser shot is directed to the tip of the flagellum, which causes curling or coiling of the tail in viable sperm. Identification of viable testicular spermatozoa was shown to be comparable to that of the HOS test, and LAISS was also successfully used for selection of cryopreserved immotile sperm [67]. The fertilization rate increased significantly from 20% in the randomly selected testicular sperm group, to 45% in the laser selection group; accordingly, the take home baby rate increased from 6% to 19%. The method is quick, easy, repeatable, and safe, but it requires expensive instruments and experienced personnel [60]. LAISS has also been used successfully in PCD patients [68,69]. One study resulted in a healthy baby by LAISS-selected sperm after three unsuccessful treatment cycles, indicating the usefulness of the method in treating male infertility in PCD [69].

Based on the current knowledge, LAISS appears to be the most simple, safe, and reliable method of choice for viable sperm selection. The HOS test and sperm activation require the use of added chemicals, which may influence the embryo development. HOST and STFT are limited in selecting frozen, thawed spermatozoa, while STFT requires highly experienced personnel. When LAISS was compared to the tail flexibility test, superior reliability was concluded [60].

A high sperm DNA fragmentation rate is also strongly associated with low viability [70] and correlates with poor semen parameters (reduced count, motility, and morphology) [71,72]. In oligoasthenospermic men, it was found that 35–70% of immotile spermatozoa had poor DNA integrity in their nuclei [73]. More than 30% fragmentation rate indicates difficulty in achieving a healthy pregnancy and a higher likelihood of failed fertility treatment and miscarriage once pregnancy is achieved. Studies of DNA fragmentation in a case of repeated failed intra-cytoplasmic sperm injection (ICSI) cycles in PCD patients showed a high DNA damage rate (85%) as a possible cause of the ICSI failure [74]. The high presence of somatic cells in the ejaculate producing reactive oxygen species, may have also contributed to single-strand DNA breaks in sperm [74]. Thus, analysis of DNA fragmentation levels in PCD patients should be considered prior to ICSI to avoid unsuccessful treatment cycles and to allow for treatment accordingly with, for example, antioxidant treatment. The sperm chromatin structure assay, used to detect the DNA fragmentation index (DFI), is a routine test to estimate sperm DNA damage. Recently, a simultaneous detection of sperm membrane integrity and DNA fragmentation assay was tested using flow cytometry and co-staining, consisting of acridine orange (AO) and LIVE/DEAD™ fixable blue dead cell stain [75], to estimate sperm quality prior to selection for ART. In the case of high DNA fragmentation, testicular sperm can be selected for ICSI.

## 4. Treating Male Infertility in PCD

Diagnostic semen analysis gives insights to the likely chance of a couple conceiving naturally or by ART. In the case of subfertility or the minor decrease in normal sperm parameters, the environmental factors should also be considered in counselling patients. Observational studies have suggested that smoking, drug use, obesity, alcohol, stress, and medication are associated with poor sperm quality in men [76]. Thus, the risk factors for poor sperm quality should be eliminated prior to any other treatment considerations.

In PCD, sperm motility is often dramatically decreased and therefore ART is often required for fertilization. In vitro fertilization (IVF) or ICSI can be offered to the couple when sperm count or motility is low, but viable sperm is present in the ejaculate. ICSI is the method of choice for immotile sperm. However, poor quality samples have a reduced success rate in ICSI and therefore sperm should be carefully selected [77,78]. An increased number of sperm with poor morphology does not seem to reduce IVF or ICSI success [79], but when only abnormal sperm are present, ICSI success is decreased [80]. Normally, sperm motility is a good predictive estimate for IVF/ICSI success [78]; unfortunately, many PCD patients have totally immotile sperm. Therefore, evaluation of sperm viability is crucial for sperm selection in PCD patients. This is also important for selection against sperm DNA fragmentation and chromosomal errors, since poor quality sperm can lead to low pregnancy rates and aneuploid embryos [81].

In the case of azoospermia (a complete lack of sperm in the ejaculate), testicular sperm can be retrieved for the female partner to be used during ICSI. In men with PCD, azoospermia is rare [82,83,84], but low sperm counts (oligospermia) are often reported [57,69,74,85,86]. Sperm can be retrieved by testicular sperm aspiration (TESA), which involves aspiration of testicular tissue using needles or by surgical procedures, conventional testicular sperm extraction (cTESE), and microdissection testicular sperm extraction (mTESE). In mTESE, light microscopy is used to identify engorged seminiferous tubules, which are then dissected and inspected intraoperatively for any sperm [87]. This method is superior for the identification of viable sperm when compared to TESA and cTESE. Successful sperm retrieval has been reported in 17–45% of cTESE cases and in 45–63% of mTESE cases [88,89]. The use of testicular sperm can also be an option when the sperm viability is low in ejaculated sperm.

**Figure 4:**
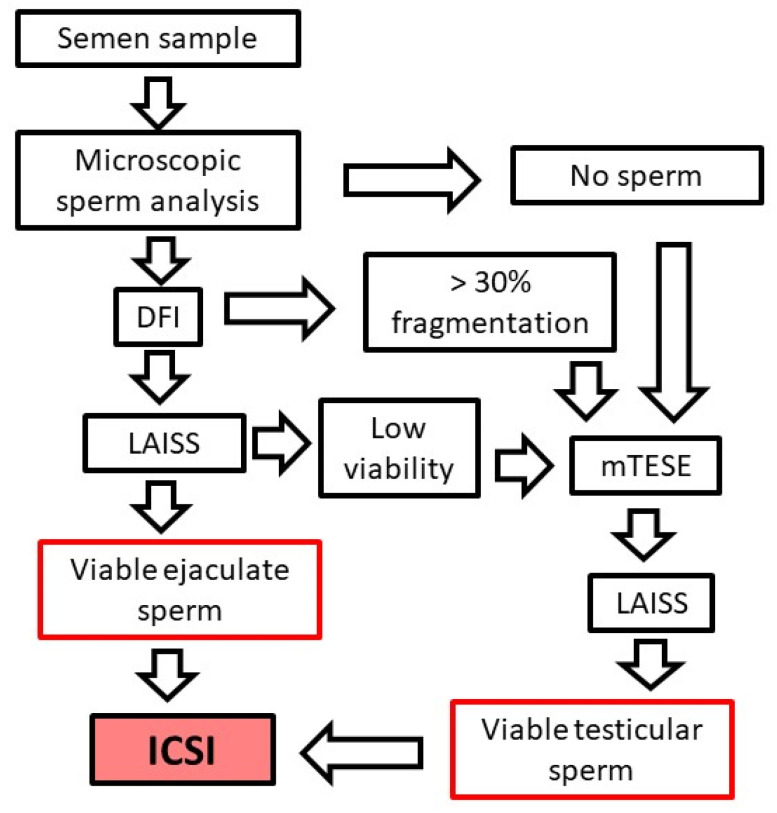
Schematic diagram of the diagnostic and treatment pipeline for male infertility in PCD. In PCD patients, a 55% fertilization rate was reported for ejaculated sperm and 65% for testicular sperm. Pregnancy rates vary between 35 and 45% for ejaculated and testicular sperm, respectively [90]. DFI = DNA fragmentation index, LAISS = laser-assisted immotile sperm selection, microdissection testicular sperm extraction (mTESE).

The treatment pathway should be decided for each patient based on the outcome of the semen analysis (Figure 4).

In the case that good quality viable sperm can be detected in the ejaculate, the selected viable sperm can be successfully used for ICSI. However, low sperm quality or count in ejaculated sperm decreases the fertilization success, and testicular sperm retrieval could be recommended (Figure 4). In all cases, patients should be informed about the possibility to pass on the PCD mutations to the next generation and the risks involved in any treatment techniques.

## 5. Success of ART in PCD Patients

The success rate of ICSI varies greatly in couples affected by PCD in the male partner. One important factor seems to be sperm viability, which can be affected by the transition time through the epididymis. As described before, motile cilia may play an important role in sperm transit through the efferent ductules to the epididymis. If this process is affected, sperm viability can decrease. Increased transport time may result in fragile sperm DNA, and hampered pronucleus formation in aged spermatozoa [58]. Furthermore, the type of ultrastructural defects may influence the rate of ICSI success as has been suggested by the association of central pair defects with lower clinical pregnancy rate [91,92]. Mutations affecting the centriolar function can prevent normal pro-nuclear development after fertilization, since the sperm centrosome is required for this process [10,93]. Thus, the ICSI success may depend on the mutated gene and its role in motile cilia and/or sperm flagellum.

Previous studies showed that patients with high sperm viability achieved reasonable fertilization rates, but patients with low sperm viability benefitted from testicular sperm retrieval [58]. Testicular sperm are generally very viable; sperm viability analysis showed that the viability difference between ejaculated and testicular sperm can be <5% and 95%, respectively [83,94]. These studies indicated that, in addition to severe oligospermia or azoospermia patients, who do not have enough sperm for ICSI, PCD patients with low sperm viability should consider testicular sperm extraction to improve treatment outcomes.

An early study evaluated the use of immotile ejaculated and testicular sperm in ICSI treatment [95]. This study showed no difference between HOS or laser selection for viable sperm, and the laser test was used for viable sperm selection for ICSI. The viability test improved fertilization success from 49 to 64% in ejaculated sperm and from 20 to 45% in testicular sperm. Live birth rates were 17 to 28% and 6 to 19% in ejaculated and testicular sperm, respectively. In PCD patients, fertilization and pregnancy rates ranging from 55 to 65% and from 35 to 45% in ejaculated and testicular sperm, respectively, were reported [90]. The overall live birth rate was estimated at 39%. Slightly better results with ejaculated sperm were reported more recently: a fertilization rate of 69% and a clinical pregnancy rate of 67% [66]. Similar estimates were reported for MMAF patients: 63–68%, 54–57%, and 43% for fertilization, pregnancy, and live birth rates, respectively [66,96]. Successful clinical pregnancies by various methods have been reported for couples with male partner PCD [57,61,62,83,84,85,86,97,98,99,100,101] and for some known PCD genes [50,66,102,103,104], as summarized in Table 3.

Isolated studies with low numbers of patients have suggested improved methods, such as ionophore application immediately after ICSI, in addition to viability and DNA fragmentation tests [86]. Although recent developments in ART have dramatically improved the prognosis of ICSI in male PCD patients, additional research is required to understand the beneficial treatment options and improve the pipeline for PCD patient fertility treatment.

## 6. Future Perspectives

PCD is a complex disease caused by mutations in a large number of genes. The genetic background is not known in all cases, but ongoing research is characterizing novel genes in PCD patients. As the effects of causative PCD mutations on male fertility have not been systematically investigated, the first objective in future studies should be the comprehensive analysis of the roles of PCD genes in spermatogenesis and sperm maturation and transport. PCD mutations can influence the sperm tail formation by altering the axoneme structure and function, as was shown for genes coding for dynein arm preassembly factors and various structural proteins. These mutations often result in immotile and even malformed sperm tails, which complicates the selection of viable sperm for ICSI. A simple and reliable test to select sperm for ICSI is yet to come, and development of a standard pipeline for fertility treatment of PCD patients should be a high priority.

In addition to sperm tail formation, PCD genes may affect male fertility through efferent duct motile cilia. The importance of motile ED cilia was recently recognized, but the role of this cilia type is not understood in PCD patients. Interestingly, fertility and infertility was reported for genes not expressed during spermatogenesis in PCD patients. Furthermore, azoospermia was detected in few PCD patients, indicating a problem in sperm transport. It can be speculated that the variable sperm count in PCD patients is due to the malfunction of male-specific motile cilia. The role of ED cilia, and causes of variable fertility status, need to be studied in detail and may enable the development of novel treatment options for patients. Since defects in cilia motility may influence sperm transport and maturation, leading to quality issues, these processes should be understood to improve male factor fertility.

## Figures and Tables

**Table 1:** Classification of sperm phenotypes.

Defect	Sperm Phenotype
Normozoospermia	Normal ejaculate and semen parameters
Azoospermia	No sperm in the ejaculate
Oligozoospermia	Sperm concentration <15 million/mL
Asthenozoospermia	Sperm motility <40%
Teratozoospermia	Normal morphology <4%
Oligoasthenozoospermia	Sperm concentration <15 million/mL
	Sperm motility <40%
Oligoteratozoospermia	Sperm concentration <15 million/mL
	Normal morphology <4%
Asthenoteratozoospermia	Sperm motility <40%
	Normal morphology <4%
Oligoasthenoteratozoospermia	Sperm concentration <15 million/mL
	Sperm motility <40%
	Normal morphology <4%

**Table 2:** Normal sperm quality parameters based on WHO guidelines.

	WHO Reference Range
Total sperm count in ejaculate	39–928 million
Ejaculate volume	1.5–7.6 mL
Sperm concentration	15–259 million per mL
Total motility (progressive and non-progressive)	40–81 percent
Progressive motility	32–75 percent
Sperm morphology	4–48 percent

**Table 3:** Reported ICSI treatments and outcomes in PCD patients.

Gene	ICSI Result	Sperm Origin	Reported Sperm Phenotype	Viability %	Sperm Count	Reference
-	live birth	ejaculated sperm	immotile	90	normal	[97]
-	live birth	ejaculated sperm, swim-up	25% motility	nd	normal	[98]
-	live birth	testicular sperm	azoospermia	nd	0	[83]
-	live birth	testicular sperm	immotile	<5%	low (4.8)	[83]
-	live birth	testicular sperm	immotile, severe oligospermia	nd	extremely low	[57]
-	pregnancy	testicular sperm	immotile	>65	normal	[57]
-	live birth	testicular sperm	immotile, malformed	nd	normal	[94]
-	live birth	viable ejaculated sperm	immotile, dynein arm defect	40	normal	[61]
-	live birth	ejaculated sperm, swim-up	0.3% motile, lack of dynein arms	30	normal	[99]
-	no pregnancy	viable ejaculated and testicular	0.3% motile, 76.4% DNA fragmentation	30	low (1.2)	[74]
-	live birth	viable ejaculated sperm	immotile, dynein arm defect	54	low (0.9)	[85]
-	live birth	testicular sperm	azoospermia	nd	0	[84]
-	live birth	viable ejaculated sperm	immotile	nd	normal	[62]
-	live birth	testicular sperm	immotile, malformed	20	low (10.1)	[100]
-	live birth	viable ejaculated sperm,	immotile, abnormal morphology	32	low (1.8)	[86]
-	live birth	viable ejaculated sperm	immotile	60–74	normal	[101]
-	no pregnancy	viable ejaculated sperm	immotile	35–65	normal	[101]
ZMYND10	healthy baby	viable ejaculated sperm	immotile, oligoasthenoteratozoospermia, lack of dynein arms	54	low (2–9)	[69]
CFAP74	healthy baby	ejaculated sperm	low motility (2%), malformed	nd	normal	[103]
DNAAF6	healthy baby	viable ejaculated sperm	immotile	nd	low (5.2–11.2)	[104]
DNAAF7	healthy baby	viable ejaculated sperm	low motility (8.6%)	nd	low (3.5–8.7)	[104]
SPAG6	healthy baby	viable ejaculated sperm	immotile, malformed	nd	normal	[66]
RSPH3	healthy baby	viable ejaculated sperm	immotile, malformed	nd	normal	[66]
DNAH9	clinical pregnancy	motile ejaculated sperm	very low motility (0.3–0.8% motile)	75–82	normal	[50]
LRRC6	clinical pregnancy	testicular sperm	immotile, axoneme defects	75	normal	[102]

Live birth = no data about the health of the baby reported.
